# Ethical aspects of brain death and end-of-life

**DOI:** 10.1590/S1980-57642008DN10300002

**Published:** 2007

**Authors:** Gabriel Oselka, Reinaldo Ayer de Oliveira

**Affiliations:** 1 Associate Professor, Department of Pediatrics and Department of Legal Medicine, Medical Ethics and Social and Labor Medicine, São Paulo University Faculty of Medicine.; 2 Assistant Doctor, Department of Legal Medicine, Medical Ethics and Social and Labor Medicine, São Paulo University Faculty of Medicine. Member of the Regional Medical Council of the State of São Paulo.

**Keywords:** brain death, end-of-life, ethics

## Abstract

Ethical issues surrounding brain death and end-of-life have not been afforded in Brazil the same attention as in many developed countries. There appears to be reluctance on the part of Brazilian doctors to limit or suspend procedures or treatment which prolongs life of patients in terminal phases of severe incurable illness, or to suspend the artificial means of supporting vegetative functions in cases of brain death outside the context of organ and tissue donation for transplant. Fears grounded in possible administrative (Regional Medical Councils) or legal repercussions, as well as ambiguous interpretations of religious precepts, partially explain this reluctance which often results in unnecessary prolonging of patient suffering. A recent resolution by the Federal Medical Council on end-of-life may offer doctors some guidance and confidence in dealing with highly complex ethical situations.

## Abstract (pt)

Questões éticas relativas à morte encefálica e terminalidade da vida não têm ainda no Brasil o mesmo destaque que em países desenvolvidos. Os médicos brasileiros parecem ter resistência em limitar ou suspender tratamentos ou procedimentos de prolongamento da vida em pacientes em fase terminal de doença grave e incurável, ou em suspender os meios artificiais da manutenção de funções vegetativas em casos de morte encefálica fora do contexto de doação de órgãos e tecidos para transplante. Receio quanto a possíveis sanções administrativas (Conselhos Regionais de Medicina) ou legais, além de duvidosas interpretações de preceitos religiosos explicam, ao menos parcialmente, essa relutância, que freqüentemente resulta em prolongamento desnecessário do sofrimento dos pacientes. Uma recente Resolução do Conselho Federal de Medicina sobre terminalidade de vida oferece aos médicos orientação sobre como lidar com algumas dessas questões de grande complexidade ética.

Any discussion on brain death must take into account the first Brazilian law on transplants which clearly stated that the criteria for defining brain death were to be determined by the Federal Medical Council (FMC). This law was promulgated in February 1997 and states that removal *post mortem*, of tissues, organs or parts of the human body destined for transplant or treatment must be preceded by diagnosed brain death, according to the clinical and technological criteria set forth by resolution of the Federal Medical Council.^1^

Normative Resolutions are the manner in which the Regional and Federal Medical Councils establish laws to complement the Medical Ethics Code. A pertinent comparison is that the Medical Ethics Code is equivalent to the Federal Constitution while the Councils’ Normative Resolutions are equivalent to common law: in this context, doctors are obliged to abide by these laws.

The FMC enacted a Resolution in 1997 which, upon establishing the criteria for brain death took into consideration that:

- Total and irreversible arrest of brain functions are equivalent to death, according to well established criteria by the global scientific community;
- There is a psychological and material burden caused by prolonging the use of extraordinary resources to support vegetative functions in patients with total and irreversible arrest of brain activity;
- Judicious indication is required for withdrawing these resources;
- There is a need to adopt criteria to indisputably ascertain occurrence of death.

This resolution set forth that: “Brain death shall be characterized by performing clinical and complementary exams during variable time intervals established according to age bracket”. The Resolution also stipulates that brain death must have resulted from an irreversible process and a known cause, and that the clinical parameters to be observed in ascertaining brain death are: aperceptive coma with absence of supra-spinal motor activity and apnea.^2^

Enactment of this resolution created a rare situation in Brazil whereby a clear definition by the organ responsible for medical ethics on what constitutes brain death exists in parallel with a legal position establishing brain death to be that determined by the FMC. This is an exceptional situation amidst the numerous ethical dilemmas in our milieu. For example, assisted reproduction also has a normative resolution by the FMC, but ethical debates on the issue remain extremely heated, yet no laws provide for norms governing procedures related to assisted reproduction. Likewise, issues surrounding end-of-life have no specific legislation, and as will become apparent, the ethical discussion on this matter is also heated.

Despite the evident ethical and legal agreement regarding brain death, in practice these cases continue to be a source of doubt and controversy in Brazil. In the context of organ donation for organ and tissue transplant this presents no great obstacle: the law and the resolution are properly applied and do not give rise to problems. However, when brain death is ascertained outside the organ donation context, the situation is radically different. In such cases, ascertainment of brain death is often not followed by suspension of ventilation support and other life-prolonging measures. For instance, a recent study (3) within Pediatric Intensive Care Units (PICUs) revealed that the time period for withdrawal of vital support following brain death was 1.8±1.9hs, 28.6±43.2 hs and 15.5±17.1 hs in PICUs located in Southern, Southeastern and Northeastern regions, respectively. In 41% of cases in the Southeastern region and 44% in the Northeastern region, vital support was maintained for more than 24 hours after brain death had been diagnosed.

Thus there seems to be a curious dichotomy among Brazilian doctors, with clear acceptance of the criteria in cases of organ donation for organ transplants, yet reluctance to accept them in other situations. The FMC has been consulted on more than one occasion regarding this issue and has reiterated in a report that “when a patient is considered brain dead, and therefore deceased, the doctor responsible for the patient, prior to suspending artificial means of sustaining vegetative functions, must notify the family to allow time for them to question the diagnosis, since this practice has not yet been incorporated into the culture of the people..” On the other hand, it also emphasized that “from this point forth, prolonging care constitutes unjustifiable therapeutic obstinacy, providing no benefit to patient or their family”. Should the family refuse to accept withdrawal of care, doctors in charge of the patient may still carry this out as “verification of death by any criteria is the doctor’s remit”. However, doctors should exercise sensitivity such that these powers do not constitute additional cause of pain to relatives already suffering the loss of a loved one, who should greet a message of relief and solidarity in the doctor”^4^.

This scenario, which is definitely not restricted to only pediatric PICUs, is cause for concern, and the FMC is working on the enactment of a normative resolution which will contribute toward definitively clarifying that criteria for brain death are valid in all situations, not being dependent on possible organ and tissue donation.

Another situation linked to end-of-life raises even more complex questions. There are a vast number of patients, both children and adults, hospitalized in infirmaries or intensive care units, in a terminal phase of severe and incurable illness, who are being given life-prolonging treatment, without these individuals having been given the option to limit this type of treatment. According to a description by Kipper, “these are often patients submitted to technological paraphernalia which are not only unable to relieve their pain and suffering, but also prolong and increase it unnecessarily; these are human beings submitted to therapeutic obstinacy, disproportionate treatment or futile medical practices”.^5^

This situation is not peculiar to Brazil, it does however appear to be more common among us than in many developed countries.^6-8^ Why should this be the case? The answer is not straightforward. Certainly, there are a series of contributing factors, but three seem to be fundamental; fear of judicial repercussions, fear of administrative consequences in the Medical Council ambit, and religious beliefs. We shall examine each briefly.

Many doctors believe that the Medical Ethics Code^9^ takes an opposing stance to any manner of restricting treatment to terminal patients. Specifically, they cite article 57 of the Code, which states that doctors are prohibited from “not using all means of diagnosis and treatment available to them to benefit the patient”. Having participated directly in the process of drafting this Code of 1988, one of the authors (GO) assures that the emphasis of this article is the use of available treatment *to benefit the patient*, which in turn does not imply *always* using all treatments available. The key point is to assess – and this is a joint decision between the doctor and the patients or those representing them – over whether a given intervention prolonging life in that case will benefit the patient.

Clearly, however, the manner in which the article is worded leads to ambiguous interpretation. For this reason, a recent FMC resolution on end-of-life is so crucial.^10^ The council’s position is clearly emphasized in the 1st article which states: “Doctors are permitted to limit or suspend procedures and treatment which prolongs life of patients in a terminal phase of a severe and incurable illness, respecting the person’s will or that of their legal representative”.

There are also other important aspects addressed in the resolution. In addition to noting that “The decision ... must have grounds and be recorded on medical records”, it highlights that the patient shall continue receiving all care needed to alleviate symptoms which lead to suffering, and ensure full care, psychic, social and spiritual comfort, as well as guaranteeing the right to hospital discharge”.

Moreover, it is noteworthy that no cases have been brought before the Medical Council where the doctor having decided, in agreement with the patient or his/her legal representative to limit or suspend treatment of patients in the terminal phase, has been prosecuted and sentenced for this conduct.

Fear of legal repercussions of suspending or limiting treatment is a more complex discussion. Unfortunately, in contrast to what has been the case in developed countries for decades, Brazil has no clear jurisprudence on this matter, leaving this open to different interpretations. There are a number of respected attorneys and jurists who defend the notion that any suspension or limitation of treatment, even in patients clearly incapable of recovering, constitutes dereliction of medical duty, with all the legal consequences this implies. This is apparently the perception of a large proportion of Brazilian doctors. Fortunately however, the position of other attorneys and jurists has prevailed in recent years, such as Paulo Daher Rodrigues, who deems that “when a doctor interrupts therapeutic care because it is of no further aid, there is no legal obligation to act and no grounds for punishment”. Elida Sá, also a jurist, affirms that “omission (of the doctor) does not characterize a criminal act, given the absence of legal duty, if good health was unattainable”. Finally, Paulo José da Costa Júnior points out that “there is no legal obligation to prolong an unrecoverable life”.^11^

A São Paulo State law of March 1999, whose constitutional grounds have never been called into question, provides for patient rights, where one of its articles states the patient has the right to “refuse painful or extraordinary treatment to prolong life” while another states “the patient has the right to choose where they are to die”.^12^

Again, to date there have been no cases in common courts of a doctor being denounced, sued or sentenced for this reason. Although ideally the issue should be definitively clarified, we believe that if the doctor’s conscience indicates that the best course for a patient in the terminal phase of severe and incurable illness is to withdraw or limit treatment, provided the patient agrees, there is no legal obstacle to this conduct.

Occasionally there are objections to limiting treatment in terminal patients for religious reasons. This is particularly true in Brazil with regard to the position of the Catholic Church, recognized as an intransigent advocate of the sacredness of life. Doctors and patients often believe the Catholic Church would never accept any manner of treatment limitation, on the understanding that this is a Divine prerogative. However, since Pope Pius XII it recognizes that “it is valid in conscience to take the decision to refuse treatment which would lead only to precarious and painful prolonging of life, without however withdrawing other normal care given to patients in similar cases”. In one of his encyclicals, John Paul II, lucidly and in-depth, clarifies the position with which many patients and doctors certainly agree: “refusal of extraordinary or disproportionate means [of prolonging life] do not amount to suicide or euthanasia, and is primarily acceptance of the human condition concerning death”.^13^

Attempting to understand the factors behind doctors not offering patients in terminal phases of severe and incurable sickness the options the doctors deem best, constitutes part of the absolutely indispensable process of involving not only doctors and other health professionals, but also society as a whole, in discussing a situation which is currently clearly not working in the best interests of our patients.
